# (3*S*,12*R*,20*S*,24*R*)-20,24-Ep­oxy­dammarane-3,12,25-triol

**DOI:** 10.1107/S1600536811028418

**Published:** 2011-07-23

**Authors:** Wen-Juan Li, Huan-Mei Guo, Chun-Mei Ji, Yi Bi, Qing-Guo Meng

**Affiliations:** a School of Pharmacy, Yantai University, Yantai 264005, People’s Republic of China; b Microscale Science Institute, Weifang University, Weifang 261041, People’s Republic of China; c Weifang People’s Hospital, Weifang 261041, People’s Republic of China

## Abstract

In the title mol­ecule, C_30_H_52_O_4_, the three six-membered rings are in chair conformations, the cyclo­pentane ring is in an envelope form and the tetra­hydro­furan ring has a conformation inter­mediate between half-chair and sofa. In the crystal, mol­ecules are linked by inter­molecular O—H⋯O hydrogen bonds into helical chains along [100]. Two intra­molecular O—H⋯O hydrogen bonds are also present. One C atom of the tetrahydrofuran ring and its attached H atoms are equally disordered over two sets of sites.

## Related literature

For the medicinal properties of *Panax ginseng* and *Panax quinquefolium*, see: Shibata *et al.* (1985 ▶); Takano *et al.* (1999 ▶); Yu *et al.* (2007 ▶); Wang *et al.* (2010 ▶). For related structures, see: Guo *et al.* (2011 ▶); Iljin *et al.* (1982 ▶); Meng *et al.* (2010 ▶).
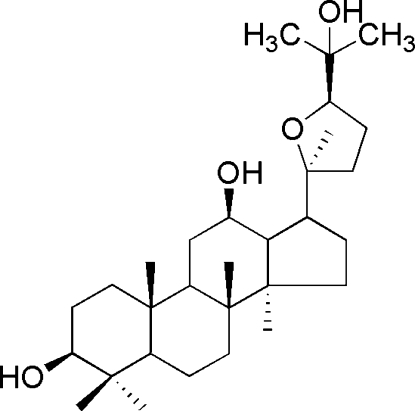


## Experimental

### 

#### Crystal data

C_30_H_52_O_4_
*M* *_r_* = 476.72
Orthorhombic, 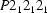

*a* = 7.6795 (14) Å
*b* = 13.067 (3) Å
*c* = 28.084 (5) Å
*V* = 2818.1 (9) Å^3^
*Z* = 4
Mo *K*α radiation
μ = 0.07 mm^−1^
*T* = 298 K
0.20 × 0.20 × 0.16 mm

#### Data collection

Bruker SMART CCD diffractometer
14876 measured reflections
5250 independent reflections
3460 reflections with *I* > 2σ(*I*)
*R*_int_ = 0.048

#### Refinement

*R*[*F* ^2^ > 2σ(*F* ^2^)] = 0.062
*wR*(*F* ^2^) = 0.128
*S* = 1.06
5250 reflections
318 parameters
H-atom parameters constrained
Δρ_max_ = 0.15 e Å^−3^
Δρ_min_ = −0.20 e Å^−3^

### 

Data collection: *SMART* (Bruker, 1997 ▶); cell refinement: *SAINT* (Bruker, 1997 ▶); data reduction: *SAINT*; program(s) used to solve structure: *SHELXS97* (Sheldrick, 2008 ▶); program(s) used to refine structure: *SHELXL97* (Sheldrick, 2008 ▶); molecular graphics: *SHELXTL* (Sheldrick, 2008 ▶); software used to prepare material for publication: *SHELXTL*.

## Supplementary Material

Crystal structure: contains datablock(s) global, I. DOI: 10.1107/S1600536811028418/lh5260sup1.cif

Structure factors: contains datablock(s) I. DOI: 10.1107/S1600536811028418/lh5260Isup2.hkl

Additional supplementary materials:  crystallographic information; 3D view; checkCIF report

## Figures and Tables

**Table 1:** Hydrogen-bond geometry (Å, °)

*D*—H⋯*A*	*D*—H	H⋯*A*	*D*⋯*A*	*D*—H⋯*A*
O4—H4*A*⋯O1^i^	0.82	2.09	2.905 (3)	172
O3—H3⋯O2	0.82	1.95	2.677 (3)	147
O1—H1⋯O3	0.82	2.14	2.948 (3)	170

Symmetry code: (i) 
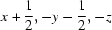
.

**Fig. 1.**
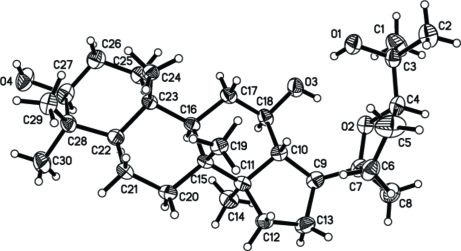
The molecular structure of the title compound. Displacement ellipsoids are drawn at the 30% probability level.

## supplementary crystallographic information

### Comment

Both Panax ginseng and Panax quinquefolium, belonging to the Araliaceae, are well known traditional medicinal herbs. They are used as tonics and the treatment for diseases, such as tumor and myocardial ischemia. Panax ginseng contains numbers of ginsenoside, including an oleanolic acid-type saponin in addition to the major protopanaxadiol and protopanaxatriol-type saponins (Shibata *et al.*, 1985). Panax quinquefolium contains an ocotillol-type (20*S*, 24*R*-epoxyside) saponin with high anti-tumor activity (Takano *et al.*, 1999), as well as an oleanolic acid-type saponin, protopanaxadiol and protopanaxatriol-type saponins. (3*S*,6S,12*R*,20S,24*R*)-20,24-epoxy-dammarane-3,6,12,25-tetraol and (3*S*,12*R*,20*S*,24*R*)-20,24-epoxy-dammarane-3,12,25-triol are found to possess cardioprotective effect on myocardial injury induced by isoproterenol in rats (Yu *et al.*, 2007; Wang *et al.*, 2010). As part of our ongoing investigation of ocotillol-type compounds and their cardioprotective effect on myocardial injury, we report herein the crystal structure of the title compound, (I).

In the molecule (Fig. 1), all bond lengths and angles are within normal ranges (Guo *et al.*, 2011; Iljin *et al.*, 1982; Meng *et al.*, 2010) Rings A(C10/C11/C15-C18), B(C15/C16/C20-C23), and C(C22/C23/C25-C28) are in chair conformations. Ring D(C9-C13) has an envelope form with C11 as the flap. The tetrahydrofuran ring has a conformation intermediate between the half-chair and envelope forms. In the crystal, molecules are linked by intermolecular O—H···O hydrogen bonds into helical chains along [100]. Two intramolecular O—H···O hydrogen bonds are also present.

### Experimental

20(S)-protopanaxadiol was degraded from Panax quinquefolium saponin with sodium methylate in DMSO at about 463-473K and seperated by silica colum chromatography. (3*S*,12*R*,20*S*,24*R*)-20,24-epoxy-dammarane-3,12,25-triol was synthesized from 20(S)-protopanaxadiol in the presence of *N*,*N*-dimethylaminopyridine, pyridine and acetic anhydride. The intermediate esters were oxidized by hydrogen dioxide 30% solution in methanoic acid, and the title compound was prepared by saponification with sodium hydroxide in methanol and seperated by silica colum chromatography. Finally, the crystals were dried at room temperature the title compound was crystallized from ethyl acetate and petroleum ether. Single crystals of compound (I) suitable for X-ray measurements were obtained by recrystallization from ethyl acetate and petroleum ether at room temperature.

### Refinement

All H atoms were fixed geometrically and allowed to ride on their attached atoms, with C—H distances in the range 0.93–0.98 Å, and with *U*_iso_(H) = 1.2*U*_eq_(C) or *U*_iso_(H) = 1.5*U*_eq_(C_methyl_). The absolute configuration is based on unchanging stereochemical centers in the synthesis.

### Crystal data

**Table:**

C_30_H_52_O_4_	*F*(000) = 1056
*M**_r_* = 476.72	*D*_x_ = 1.124 Mg m^−^^3^
Orthorhombic, *P*2_1_2_1_2_1_	Mo *K*α radiation, λ = 0.71073 Å
Hall symbol: P 2ac 2ab	Cell parameters from 2282 reflections
*a* = 7.6795 (14) Å	θ = 2.7–20.8°
*b* = 13.067 (3) Å	µ = 0.07 mm^−^^1^
*c* = 28.084 (5) Å	*T* = 298 K
*V* = 2818.1 (9) Å^3^	Block, colourless
*Z* = 4	0.20 × 0.20 × 0.16 mm

### Data collection

**Table:**

Bruker SMART CCD diffractometer	3460 reflections with *I* > 2σ(*I*)
Radiation source: fine-focus sealed tube	*R*_int_ = 0.048
graphite	θ_max_ = 25.5°, θ_min_ = 1.7°
φ and ω scans	*h* = −8→9
14876 measured reflections	*k* = −15→13
5250 independent reflections	*l* = −34→34

### Refinement

**Table:**

Refinement on *F*^2^	Primary atom site location: structure-invariant direct methods
Least-squares matrix: full	Secondary atom site location: difference Fourier map
*R*[*F*^2^ > 2σ(*F*^2^)] = 0.062	Hydrogen site location: inferred from neighbouring sites
*wR*(*F*^2^) = 0.128	H-atom parameters constrained
*S* = 1.06	*w* = 1/[σ^2^(*F*_o_^2^) + (0.0537*P*)^2^] where *P* = (*F*_o_^2^ + 2*F*_c_^2^)/3
5250 reflections	(Δ/σ)_max_ < 0.001
318 parameters	Δρ_max_ = 0.15 e Å^−^^3^
0 restraints	Δρ_min_ = −0.20 e Å^−^^3^

### Special details

**Table:**

Geometry. All e.s.d.'s (except the e.s.d. in the dihedral angle between two l.s. planes) are estimated using the full covariance matrix. The cell e.s.d.'s are taken into account individually in the estimation of e.s.d.'s in distances, angles and torsion angles; correlations between e.s.d.'s in cell parameters are only used when they are defined by crystal symmetry. An approximate (isotropic) treatment of cell e.s.d.'s is used for estimating e.s.d.'s involving l.s. planes.
Refinement. Refinement of *F*^2^ against ALL reflections. The weighted *R*-factor *wR* and goodness of fit *S* are based on *F*^2^, conventional *R*-factors *R* are based on *F*, with *F* set to zero for negative *F*^2^. The threshold expression of *F*^2^ > σ(*F*^2^) is used only for calculating *R*-factors(gt) *etc*. and is not relevant to the choice of reflections for refinement. *R*-factors based on *F*^2^ are statistically about twice as large as those based on *F*, and *R*- factors based on ALL data will be even larger.

### Fractional atomic coordinates and isotropic or equivalent isotropic displacement parameters (Å^2^)

**Table:**

	*x*	*y*	*z*	*U*_iso_*/*U*_eq_	Occ. (<1)
C1	0.4251 (4)	−0.0332 (3)	0.21114 (13)	0.0847 (12)	
H1A	0.4198	0.0041	0.1817	0.127*	
H1B	0.3868	0.0099	0.2367	0.127*	
H1C	0.3511	−0.0923	0.2092	0.127*	
C2	0.6196 (5)	−0.1359 (3)	0.26373 (10)	0.0736 (11)	
H2A	0.5505	−0.1960	0.2584	0.110*	
H2B	0.5759	−0.0998	0.2910	0.110*	
H2C	0.7384	−0.1554	0.2693	0.110*	
C3	0.6101 (4)	−0.0668 (2)	0.22022 (10)	0.0507 (8)	
C4	0.7278 (4)	0.0254 (3)	0.22727 (10)	0.0558 (9)	
H4	0.6876	0.0642	0.2550	0.067*	
C5	0.9203 (5)	0.0014 (3)	0.23315 (15)	0.1004 (14)	0.50
H5A	0.9496	−0.0060	0.2666	0.120*	0.50
H5B	0.9498	−0.0617	0.2168	0.120*	0.50
C5'	0.9203 (5)	0.0014 (3)	0.23315 (15)	0.1004 (14)	0.50
H5'1	0.9496	−0.0060	0.2666	0.120*	0.50
H5'2	0.9498	−0.0617	0.2168	0.120*	0.50
C6	1.0160 (4)	0.0894 (3)	0.21188 (11)	0.0649 (10)	
H6A	1.1036	0.0655	0.1896	0.078*	
H6B	1.0729	0.1293	0.2365	0.078*	
C7	0.8793 (4)	0.1540 (2)	0.18612 (11)	0.0528 (8)	
C8	0.8352 (5)	0.2515 (3)	0.21402 (12)	0.0794 (11)	
H8A	0.7426	0.2874	0.1983	0.119*	
H8B	0.9362	0.2947	0.2156	0.119*	
H8C	0.7992	0.2335	0.2457	0.119*	
C9	0.9254 (4)	0.1805 (2)	0.13455 (10)	0.0510 (8)	
H9	0.8368	0.2274	0.1222	0.061*	
C10	0.9449 (3)	0.0904 (2)	0.09921 (9)	0.0418 (7)	
H10	0.9894	0.0319	0.1173	0.050*	
C11	1.0907 (3)	0.1237 (2)	0.06455 (10)	0.0411 (7)	
C12	1.2203 (4)	0.1710 (2)	0.09937 (10)	0.0523 (8)	
H12A	1.3044	0.2134	0.0828	0.063*	
H12B	1.2819	0.1184	0.1170	0.063*	
C13	1.1069 (4)	0.2358 (2)	0.13266 (11)	0.0635 (9)	
H13A	1.0944	0.3048	0.1204	0.076*	
H13B	1.1582	0.2393	0.1642	0.076*	
C14	1.0295 (4)	0.2132 (2)	0.03248 (11)	0.0607 (9)	
H14A	0.9573	0.2587	0.0507	0.091*	
H14B	0.9643	0.1866	0.0061	0.091*	
H14C	1.1291	0.2498	0.0208	0.091*	
C15	1.1547 (3)	0.0288 (2)	0.03503 (9)	0.0360 (6)	
C16	0.9957 (3)	−0.0086 (2)	0.00506 (8)	0.0389 (7)	
H16	0.9580	0.0524	−0.0124	0.047*	
C17	0.8403 (3)	−0.0340 (2)	0.03794 (10)	0.0480 (8)	
H17A	0.7410	−0.0521	0.0183	0.058*	
H17B	0.8700	−0.0935	0.0570	0.058*	
C18	0.7877 (3)	0.0529 (2)	0.07120 (10)	0.0464 (7)	
H18	0.7421	0.1096	0.0521	0.056*	
C19	1.2256 (3)	−0.0554 (2)	0.06845 (10)	0.0435 (7)	
H19A	1.2910	−0.1042	0.0502	0.065*	
H19B	1.1301	−0.0893	0.0839	0.065*	
H19C	1.2997	−0.0250	0.0921	0.065*	
C20	1.3025 (4)	0.0595 (2)	0.00172 (10)	0.0513 (8)	
H20A	1.2722	0.1236	−0.0136	0.062*	
H20B	1.4066	0.0713	0.0205	0.062*	
C21	1.3433 (3)	−0.0191 (2)	−0.03635 (10)	0.0498 (8)	
H21A	1.3851	−0.0815	−0.0215	0.060*	
H21B	1.4346	0.0067	−0.0569	0.060*	
C22	1.1812 (4)	−0.0429 (2)	−0.06606 (9)	0.0452 (7)	
H22	1.1364	0.0246	−0.0750	0.054*	
C23	1.0341 (3)	−0.0895 (2)	−0.03472 (9)	0.0394 (7)	
C24	1.0780 (4)	−0.1959 (2)	−0.01420 (9)	0.0462 (8)	
H24A	1.0064	−0.2089	0.0132	0.069*	
H24B	1.1984	−0.1977	−0.0051	0.069*	
H24C	1.0565	−0.2472	−0.0379	0.069*	
C25	0.8724 (4)	−0.1010 (2)	−0.06637 (10)	0.0535 (8)	
H25A	0.7823	−0.1361	−0.0485	0.064*	
H25B	0.8289	−0.0334	−0.0744	0.064*	
C26	0.9066 (4)	−0.1596 (3)	−0.11209 (10)	0.0617 (9)	
H26A	0.9409	−0.2291	−0.1044	0.074*	
H26B	0.8003	−0.1629	−0.1307	0.074*	
C27	1.0486 (5)	−0.1094 (3)	−0.14150 (9)	0.0606 (9)	
H27	1.0068	−0.0402	−0.1485	0.073*	
C28	1.2207 (4)	−0.0958 (2)	−0.11462 (10)	0.0515 (8)	
C29	1.3187 (4)	−0.1977 (3)	−0.10958 (11)	0.0630 (9)	
H29A	1.3576	−0.2201	−0.1404	0.095*	
H29B	1.2423	−0.2483	−0.0962	0.095*	
H29C	1.4174	−0.1885	−0.0890	0.095*	
C30	1.3365 (5)	−0.0241 (3)	−0.14445 (11)	0.0851 (13)	
H30A	1.3411	−0.0484	−0.1767	0.128*	
H30B	1.4519	−0.0230	−0.1313	0.128*	
H30C	1.2887	0.0438	−0.1439	0.128*	
O1	0.6710 (4)	−0.12800 (17)	0.18183 (7)	0.0698 (7)	
H1	0.6729	−0.0937	0.1574	0.105*	
O2	0.7239 (2)	0.09040 (15)	0.18631 (6)	0.0488 (5)	
O3	0.6511 (3)	0.01346 (19)	0.10025 (7)	0.0650 (7)	
H3	0.6383	0.0504	0.1236	0.097*	
O4	1.0687 (4)	−0.15820 (18)	−0.18635 (7)	0.0770 (7)	
H4A	1.0998	−0.2176	−0.1823	0.115*	

### Atomic displacement parameters (Å^2^)

**Table:**

	*U*^11^	*U*^22^	*U*^33^	*U*^12^	*U*^13^	*U*^23^
C1	0.056 (2)	0.111 (3)	0.087 (2)	0.004 (2)	0.007 (2)	0.025 (2)
C2	0.075 (2)	0.087 (3)	0.0594 (19)	0.008 (2)	0.0038 (19)	0.009 (2)
C3	0.0429 (17)	0.064 (2)	0.0448 (16)	0.0055 (17)	0.0008 (15)	−0.0015 (17)
C4	0.066 (2)	0.062 (2)	0.0399 (16)	0.0061 (18)	−0.0051 (15)	−0.0050 (16)
C5	0.072 (3)	0.099 (3)	0.130 (3)	−0.009 (3)	−0.058 (3)	0.024 (3)
C5'	0.072 (3)	0.099 (3)	0.130 (3)	−0.009 (3)	−0.058 (3)	0.024 (3)
C6	0.058 (2)	0.080 (3)	0.0566 (18)	0.005 (2)	−0.0118 (17)	−0.013 (2)
C7	0.0473 (18)	0.055 (2)	0.0561 (18)	0.0073 (16)	−0.0029 (17)	−0.0101 (17)
C8	0.082 (3)	0.066 (3)	0.090 (2)	0.001 (2)	0.019 (2)	−0.026 (2)
C9	0.0442 (18)	0.0472 (19)	0.0617 (18)	0.0097 (15)	−0.0022 (16)	−0.0082 (16)
C10	0.0372 (15)	0.0433 (17)	0.0450 (15)	0.0043 (14)	−0.0079 (13)	0.0029 (15)
C11	0.0358 (15)	0.0369 (16)	0.0507 (16)	0.0018 (14)	−0.0012 (14)	0.0071 (14)
C12	0.0475 (18)	0.0480 (19)	0.0614 (18)	−0.0089 (15)	0.0041 (16)	−0.0069 (16)
C13	0.061 (2)	0.051 (2)	0.078 (2)	−0.0041 (18)	−0.0013 (19)	−0.0137 (19)
C14	0.062 (2)	0.0457 (19)	0.075 (2)	0.0087 (17)	0.0024 (18)	0.0109 (18)
C15	0.0314 (14)	0.0343 (16)	0.0423 (14)	−0.0033 (13)	−0.0005 (12)	0.0082 (14)
C16	0.0377 (16)	0.0377 (16)	0.0412 (14)	−0.0032 (13)	−0.0020 (13)	0.0103 (14)
C17	0.0356 (16)	0.061 (2)	0.0468 (16)	−0.0084 (15)	−0.0057 (14)	0.0044 (16)
C18	0.0306 (15)	0.064 (2)	0.0442 (15)	0.0011 (15)	−0.0011 (14)	0.0065 (16)
C19	0.0383 (15)	0.0446 (17)	0.0478 (15)	0.0022 (14)	−0.0060 (14)	−0.0002 (14)
C20	0.0465 (18)	0.0463 (19)	0.0612 (18)	−0.0102 (15)	0.0022 (16)	0.0030 (17)
C21	0.0440 (17)	0.0532 (19)	0.0523 (17)	−0.0088 (16)	0.0101 (15)	0.0035 (17)
C22	0.0527 (18)	0.0391 (17)	0.0439 (15)	−0.0001 (14)	0.0001 (15)	0.0078 (14)
C23	0.0394 (15)	0.0405 (17)	0.0382 (14)	−0.0008 (14)	−0.0027 (13)	0.0064 (14)
C24	0.0552 (18)	0.0429 (18)	0.0406 (15)	−0.0081 (16)	−0.0002 (14)	0.0038 (14)
C25	0.0486 (18)	0.061 (2)	0.0509 (17)	−0.0009 (16)	−0.0091 (15)	0.0038 (17)
C26	0.063 (2)	0.073 (2)	0.0494 (17)	−0.003 (2)	−0.0159 (18)	−0.0017 (18)
C27	0.088 (3)	0.054 (2)	0.0396 (16)	0.0147 (19)	−0.0100 (18)	0.0059 (16)
C28	0.064 (2)	0.0480 (19)	0.0426 (16)	0.0005 (17)	0.0096 (16)	0.0080 (15)
C29	0.068 (2)	0.068 (2)	0.0530 (18)	0.0092 (18)	0.0011 (17)	−0.0062 (18)
C30	0.118 (3)	0.081 (3)	0.056 (2)	−0.016 (3)	0.032 (2)	0.006 (2)
O1	0.0909 (17)	0.0602 (15)	0.0584 (13)	−0.0018 (13)	0.0175 (14)	−0.0123 (12)
O2	0.0416 (11)	0.0558 (13)	0.0489 (11)	0.0111 (10)	−0.0024 (10)	0.0000 (11)
O3	0.0363 (11)	0.106 (2)	0.0525 (12)	−0.0134 (13)	0.0044 (10)	−0.0117 (13)
O4	0.118 (2)	0.0746 (17)	0.0383 (11)	0.0122 (17)	−0.0075 (13)	−0.0009 (12)

### Geometric parameters (Å, °)

**Table:**

C1—C3	1.508 (4)	C16—C17	1.545 (3)
C1—H1A	0.9600	C16—C23	1.566 (4)
C1—H1B	0.9600	C16—H16	0.9800
C1—H1C	0.9600	C17—C18	1.525 (4)
C2—C3	1.521 (4)	C17—H17A	0.9700
C2—H2A	0.9600	C17—H17B	0.9700
C2—H2B	0.9600	C18—O3	1.425 (3)
C2—H2C	0.9600	C18—H18	0.9800
C3—O1	1.421 (3)	C19—H19A	0.9600
C3—C4	1.519 (4)	C19—H19B	0.9600
C4—O2	1.430 (3)	C19—H19C	0.9600
C4—C5	1.521 (5)	C20—C21	1.516 (4)
C4—H4	0.9800	C20—H20A	0.9700
C5—C6	1.491 (5)	C20—H20B	0.9700
C5—H5A	0.9700	C21—C22	1.531 (4)
C5—H5B	0.9700	C21—H21A	0.9700
C6—C7	1.529 (4)	C21—H21B	0.9700
C6—H6A	0.9700	C22—C23	1.556 (3)
C6—H6B	0.9700	C22—C28	1.559 (4)
C7—O2	1.454 (3)	C22—H22	0.9800
C7—C9	1.530 (4)	C23—C25	1.534 (4)
C7—C8	1.533 (4)	C23—C24	1.542 (4)
C8—H8A	0.9600	C24—H24A	0.9600
C8—H8B	0.9600	C24—H24B	0.9600
C8—H8C	0.9600	C24—H24C	0.9600
C9—C10	1.547 (4)	C25—C26	1.518 (4)
C9—C13	1.571 (4)	C25—H25A	0.9700
C9—H9	0.9800	C25—H25B	0.9700
C10—C18	1.522 (3)	C26—C27	1.517 (4)
C10—C11	1.547 (4)	C26—H26A	0.9700
C10—H10	0.9800	C26—H26B	0.9700
C11—C12	1.526 (4)	C27—O4	1.420 (3)
C11—C14	1.549 (4)	C27—C28	1.533 (4)
C11—C15	1.570 (4)	C27—H27	0.9800
C12—C13	1.533 (4)	C28—C29	1.536 (4)
C12—H12A	0.9700	C28—C30	1.539 (4)
C12—H12B	0.9700	C29—H29A	0.9600
C13—H13A	0.9700	C29—H29B	0.9600
C13—H13B	0.9700	C29—H29C	0.9600
C14—H14A	0.9600	C30—H30A	0.9600
C14—H14B	0.9600	C30—H30B	0.9600
C14—H14C	0.9600	C30—H30C	0.9600
C15—C20	1.525 (3)	O1—H1	0.8200
C15—C19	1.545 (3)	O3—H3	0.8200
C15—C16	1.562 (3)	O4—H4A	0.8200
			
C3—C1—H1A	109.5	C15—C16—C23	116.6 (2)
C3—C1—H1B	109.5	C17—C16—H16	104.3
H1A—C1—H1B	109.5	C15—C16—H16	104.3
C3—C1—H1C	109.5	C23—C16—H16	104.3
H1A—C1—H1C	109.5	C18—C17—C16	114.2 (2)
H1B—C1—H1C	109.5	C18—C17—H17A	108.7
C3—C2—H2A	109.5	C16—C17—H17A	108.7
C3—C2—H2B	109.5	C18—C17—H17B	108.7
H2A—C2—H2B	109.5	C16—C17—H17B	108.7
C3—C2—H2C	109.5	H17A—C17—H17B	107.6
H2A—C2—H2C	109.5	O3—C18—C10	113.8 (2)
H2B—C2—H2C	109.5	O3—C18—C17	106.0 (2)
O1—C3—C1	110.2 (3)	C10—C18—C17	110.3 (2)
O1—C3—C4	110.4 (2)	O3—C18—H18	108.9
C1—C3—C4	110.6 (3)	C10—C18—H18	108.9
O1—C3—C2	105.1 (2)	C17—C18—H18	108.9
C1—C3—C2	110.7 (3)	C15—C19—H19A	109.5
C4—C3—C2	109.7 (2)	C15—C19—H19B	109.5
O2—C4—C3	110.7 (2)	H19A—C19—H19B	109.5
O2—C4—C5	103.3 (3)	C15—C19—H19C	109.5
C3—C4—C5	115.4 (3)	H19A—C19—H19C	109.5
O2—C4—H4	109.1	H19B—C19—H19C	109.5
C3—C4—H4	109.1	C21—C20—C15	114.1 (2)
C5—C4—H4	109.1	C21—C20—H20A	108.7
C6—C5—C4	106.0 (3)	C15—C20—H20A	108.7
C6—C5—H5A	110.5	C21—C20—H20B	108.7
C4—C5—H5A	110.5	C15—C20—H20B	108.7
C6—C5—H5B	110.5	H20A—C20—H20B	107.6
C4—C5—H5B	110.5	C20—C21—C22	110.8 (2)
H5A—C5—H5B	108.7	C20—C21—H21A	109.5
C5—C6—C7	106.1 (3)	C22—C21—H21A	109.5
C5—C6—H6A	110.5	C20—C21—H21B	109.5
C7—C6—H6A	110.5	C22—C21—H21B	109.5
C5—C6—H6B	110.5	H21A—C21—H21B	108.1
C7—C6—H6B	110.5	C21—C22—C23	111.2 (2)
H6A—C6—H6B	108.7	C21—C22—C28	114.1 (2)
O2—C7—C6	104.3 (2)	C23—C22—C28	117.6 (2)
O2—C7—C9	108.8 (2)	C21—C22—H22	104.1
C6—C7—C9	114.4 (3)	C23—C22—H22	104.1
O2—C7—C8	107.0 (2)	C28—C22—H22	104.1
C6—C7—C8	111.6 (3)	C25—C23—C24	107.8 (2)
C9—C7—C8	110.3 (3)	C25—C23—C22	107.3 (2)
C7—C8—H8A	109.5	C24—C23—C22	113.9 (2)
C7—C8—H8B	109.5	C25—C23—C16	109.1 (2)
H8A—C8—H8B	109.5	C24—C23—C16	112.5 (2)
C7—C8—H8C	109.5	C22—C23—C16	106.0 (2)
H8A—C8—H8C	109.5	C23—C24—H24A	109.5
H8B—C8—H8C	109.5	C23—C24—H24B	109.5
C7—C9—C10	117.2 (3)	H24A—C24—H24B	109.5
C7—C9—C13	109.9 (3)	C23—C24—H24C	109.5
C10—C9—C13	104.0 (2)	H24A—C24—H24C	109.5
C7—C9—H9	108.4	H24B—C24—H24C	109.5
C10—C9—H9	108.4	C26—C25—C23	113.5 (2)
C13—C9—H9	108.4	C26—C25—H25A	108.9
C18—C10—C11	109.8 (2)	C23—C25—H25A	108.9
C18—C10—C9	120.0 (2)	C26—C25—H25B	108.9
C11—C10—C9	105.1 (2)	C23—C25—H25B	108.9
C18—C10—H10	107.1	H25A—C25—H25B	107.7
C11—C10—H10	107.1	C27—C26—C25	111.5 (3)
C9—C10—H10	107.1	C27—C26—H26A	109.3
C12—C11—C10	100.5 (2)	C25—C26—H26A	109.3
C12—C11—C14	105.3 (2)	C27—C26—H26B	109.3
C10—C11—C14	111.0 (2)	C25—C26—H26B	109.3
C12—C11—C15	117.0 (2)	H26A—C26—H26B	108.0
C10—C11—C15	109.7 (2)	O4—C27—C26	111.5 (3)
C14—C11—C15	112.6 (2)	O4—C27—C28	113.3 (3)
C11—C12—C13	104.1 (2)	C26—C27—C28	113.7 (2)
C11—C12—H12A	110.9	O4—C27—H27	105.9
C13—C12—H12A	110.9	C26—C27—H27	105.9
C11—C12—H12B	110.9	C28—C27—H27	105.9
C13—C12—H12B	110.9	C27—C28—C29	111.6 (3)
H12A—C12—H12B	108.9	C27—C28—C30	107.5 (2)
C12—C13—C9	105.7 (2)	C29—C28—C30	107.2 (3)
C12—C13—H13A	110.6	C27—C28—C22	108.3 (2)
C9—C13—H13A	110.6	C29—C28—C22	113.5 (2)
C12—C13—H13B	110.6	C30—C28—C22	108.6 (2)
C9—C13—H13B	110.6	C28—C29—H29A	109.5
H13A—C13—H13B	108.7	C28—C29—H29B	109.5
C11—C14—H14A	109.5	H29A—C29—H29B	109.5
C11—C14—H14B	109.5	C28—C29—H29C	109.5
H14A—C14—H14B	109.5	H29A—C29—H29C	109.5
C11—C14—H14C	109.5	H29B—C29—H29C	109.5
H14A—C14—H14C	109.5	C28—C30—H30A	109.5
H14B—C14—H14C	109.5	C28—C30—H30B	109.5
C20—C15—C19	107.3 (2)	H30A—C30—H30B	109.5
C20—C15—C16	109.5 (2)	C28—C30—H30C	109.5
C19—C15—C16	112.4 (2)	H30A—C30—H30C	109.5
C20—C15—C11	110.4 (2)	H30B—C30—H30C	109.5
C19—C15—C11	110.6 (2)	C3—O1—H1	109.5
C16—C15—C11	106.6 (2)	C4—O2—C7	109.0 (2)
C17—C16—C15	110.42 (18)	C18—O3—H3	109.5
C17—C16—C23	115.2 (2)	C27—O4—H4A	109.5
			
O1—C3—C4—O2	63.4 (3)	C15—C16—C17—C18	54.3 (3)
C1—C3—C4—O2	−58.9 (3)	C23—C16—C17—C18	−170.9 (2)
C2—C3—C4—O2	178.7 (2)	C11—C10—C18—O3	175.7 (2)
O1—C3—C4—C5	−53.5 (4)	C9—C10—C18—O3	−62.5 (4)
C1—C3—C4—C5	−175.7 (3)	C11—C10—C18—C17	56.6 (3)
C2—C3—C4—C5	61.8 (4)	C9—C10—C18—C17	178.4 (2)
O2—C4—C5—C6	26.9 (4)	C16—C17—C18—O3	−176.7 (2)
C3—C4—C5—C6	147.8 (3)	C16—C17—C18—C10	−53.0 (3)
C4—C5—C6—C7	−9.9 (4)	C19—C15—C20—C21	73.4 (3)
C5—C6—C7—O2	−10.5 (3)	C16—C15—C20—C21	−48.8 (3)
C5—C6—C7—C9	−129.2 (3)	C11—C15—C20—C21	−165.9 (2)
C5—C6—C7—C8	104.6 (3)	C15—C20—C21—C22	56.7 (3)
O2—C7—C9—C10	−53.5 (3)	C20—C21—C22—C23	−61.8 (3)
C6—C7—C9—C10	62.6 (4)	C20—C21—C22—C28	162.3 (2)
C8—C7—C9—C10	−170.5 (3)	C21—C22—C23—C25	174.7 (2)
O2—C7—C9—C13	−172.0 (2)	C28—C22—C23—C25	−51.2 (3)
C6—C7—C9—C13	−55.9 (3)	C21—C22—C23—C24	−66.1 (3)
C8—C7—C9—C13	71.0 (3)	C28—C22—C23—C24	68.0 (3)
C7—C9—C10—C18	90.3 (3)	C21—C22—C23—C16	58.2 (3)
C13—C9—C10—C18	−148.1 (3)	C28—C22—C23—C16	−167.7 (2)
C7—C9—C10—C11	−145.5 (2)	C17—C16—C23—C25	59.1 (3)
C13—C9—C10—C11	−23.9 (3)	C15—C16—C23—C25	−169.0 (2)
C18—C10—C11—C12	172.5 (2)	C17—C16—C23—C24	−60.5 (3)
C9—C10—C11—C12	42.1 (3)	C15—C16—C23—C24	71.4 (3)
C18—C10—C11—C14	61.4 (3)	C17—C16—C23—C22	174.4 (2)
C9—C10—C11—C14	−68.9 (3)	C15—C16—C23—C22	−53.7 (3)
C18—C10—C11—C15	−63.6 (3)	C24—C23—C25—C26	−70.0 (3)
C9—C10—C11—C15	166.0 (2)	C22—C23—C25—C26	53.1 (3)
C10—C11—C12—C13	−44.1 (3)	C16—C23—C25—C26	167.5 (2)
C14—C11—C12—C13	71.4 (3)	C23—C25—C26—C27	−57.8 (3)
C15—C11—C12—C13	−162.7 (2)	C25—C26—C27—O4	−174.2 (2)
C11—C12—C13—C9	29.9 (3)	C25—C26—C27—C28	56.3 (4)
C7—C9—C13—C12	122.9 (3)	O4—C27—C28—C29	−53.7 (3)
C10—C9—C13—C12	−3.4 (3)	C26—C27—C28—C29	75.0 (3)
C12—C11—C15—C20	−64.7 (3)	O4—C27—C28—C30	63.5 (3)
C10—C11—C15—C20	−178.3 (2)	C26—C27—C28—C30	−167.8 (3)
C14—C11—C15—C20	57.6 (3)	O4—C27—C28—C22	−179.4 (2)
C12—C11—C15—C19	54.0 (3)	C26—C27—C28—C22	−50.7 (3)
C10—C11—C15—C19	−59.6 (3)	C21—C22—C28—C27	−176.9 (2)
C14—C11—C15—C19	176.3 (2)	C23—C22—C28—C27	50.2 (3)
C12—C11—C15—C16	176.5 (2)	C21—C22—C28—C29	58.6 (3)
C10—C11—C15—C16	62.9 (2)	C23—C22—C28—C29	−74.3 (3)
C14—C11—C15—C16	−61.3 (3)	C21—C22—C28—C30	−60.5 (3)
C20—C15—C16—C17	−176.6 (2)	C23—C22—C28—C30	166.7 (3)
C19—C15—C16—C17	64.2 (3)	C3—C4—O2—C7	−159.0 (2)
C11—C15—C16—C17	−57.2 (3)	C5—C4—O2—C7	−34.9 (3)
C20—C15—C16—C23	49.3 (3)	C6—C7—O2—C4	28.8 (3)
C19—C15—C16—C23	−69.9 (3)	C9—C7—O2—C4	151.3 (2)
C11—C15—C16—C23	168.8 (2)	C8—C7—O2—C4	−89.5 (3)

### Hydrogen-bond geometry (Å, °)

**Table:**

*D*—H···*A*	*D*—H	H···*A*	*D*···*A*	*D*—H···*A*
O4—H4A···O1^i^	0.82	2.09	2.905 (3)	172.
O3—H3···O2	0.82	1.95	2.677 (3)	147.
O1—H1···O3	0.82	2.14	2.948 (3)	170.

Symmetry codes: (i) *x*+1/2, −*y*−1/2, −*z*.
